# Generating Embryonic Stem Cells from the Inbred Mouse Strain DBA/2J, a Model of Glaucoma and Other Complex Diseases

**DOI:** 10.1371/journal.pone.0050081

**Published:** 2012-11-27

**Authors:** Laura G. Reinholdt, Gareth R. Howell, Anne M. Czechanski, Danilo G. Macalinao, Katharine H. MacNicoll, Chyuan-Sheng Lin, Leah Rae Donahue, Simon W. M. John

**Affiliations:** 1 The Jackson Laboratory, Bar Harbor, Maine, United States of America; 2 The Howard Hughes Medical Institute, Bar Harbor, Maine, United States of America; 3 Department of Pathology and Cell Biology, Irving Cancer Research Center, Columbia University, New York, New York, United States of America; 4 Department of Ophthalmology, Tufts University of Medicine, Boston, Massachusetts, United States of America; National Eye Institute, United States of America

## Abstract

Mouse embryonic stem (ES) cells are derived from the inner cell mass of blastocyst stage embryos and are used primarily for the creation of genetically engineered strains through gene targeting. While some inbred strains of mice are permissive to the derivation of embryonic stem cell lines and are therefore easily engineered, others are nonpermissive or recalcitrant. Genetic engineering of recalcitrant strain backgrounds requires gene targeting in a permissive background followed by extensive backcrossing of the engineered allele into the desired strain background. The inbred mouse strain DBA/2J is a recalcitrant strain that is used as a model of many human diseases, including glaucoma, deafness and schizophrenia. Here, we describe the generation of germ-line competent ES cell lines derived from DBA/2J mice. We also demonstrate the utility of DBA/2J ES cells with the creation of conditional knockout allele for Endothelin-2 (*Edn2*) directly on the DBA/2J strain background.

## Introduction

The development of gene targeting technologies and the availability of a well-annotated mouse genome sequence allows the function of individual gene(s) to be assessed in a temporal and spatial manner. Historically, the resources for genetically engineering mice have been available for a limited number of strains, including C57BL/6J (B6) and 129Sv/ImJ (129), and are more recently emerging for C57BL/6N. Resources include ES cell lines, a vast number of null and conditional alleles through the Knockout Mouse Project (KOMP) and Cre driver lines to ablate the function of individual genes in specific cell types. A major advantage of the mouse as an animal model is the availability of well-characterized inbred strains that enable functional genomics on defined genetic backgrounds. Currently, however, exploiting the full utility of mice to study human diseases is hampered by the lack of gene targeting resources for multiple inbred mouse strains.

DBA/2J is a common inbred mouse strain critical in studying a diverse range of human diseases. For example, it is widely used as an inherited model of glaucoma. Glaucoma is a neurodegenerative disorder that affects 70 million people worldwide. Clinically, no treatments that specifically target retinal ganglion cell (RGC) death are in use, as the mechanisms involved are not well understood. However, we and others have used DBA/2J extensively to identify processes involved in glaucomatous RGC death and to test potential neuroprotective treatments for human glaucoma [1], [2], [3], [4], [5], [6], [7], [8], [9], [10], [11], [12]. Secondly, DBA/2J is used to model temporal lobe epilepsy (TLE) and schizophrenia (SZ) in combination. Psychotic episodes and cognitive impairment are among the most debilitating and common comorbidities of TLE, while SZ patients are more prone to seizures than the general population. To study the overlap between both disease pathologies animal models that combine epileptic seizures with select endophenotypes of schizophrenia are essential and DBA/2J is one of the few animal models that meet these criteria [13], [14]. Finally, DBA/2J is used to model other complex diseases including deafness, cardiovascular disease and host response to infections (e.g. [15], [16], [17], [18], [19]).

Phenotypes are often very different between mouse strains with diverse genetic backgrounds and the strain characteristics of DBA/2J are often contrasted with other genetically distinct inbred strains such as C57BL/6J. These defined genetic backgrounds provide an excellent system for mapping modifier genes [20], [21], [22]. To study these differences a number of DBA/2J-relevant resources have been generated. For instance, a genome-wide panel of congenic strains has been created that contain portions of DBA/2J chromosomes on a C57BL/6J background [23]. These 65 strains contain more than 95% of the DBA/2J genome. Also, DBA/2J is one parent in two sets of recombinant inbred lines that have been generated, AKXD (the other parent being AKR/J) and BXD (the other parent being C57BL/6J). Recombinant inbred (RI) strains such as these are especially useful for mapping complex traits, since they create an immortalized mapping population that allows researchers to phenotype as many animals per genome as desired over extended periods of time. AKXD strains have been used to study a variety of diseases including murine lymphoma and glaucoma [24], [25]. The more widely used BXD strains have been used to understand the genetics of a variety of diseases and biological systems including aging, the immune system and iron regulation [26], [27], [28], [29], [30]. Much of this work has been made available through *GeneNetwork* (formerly *WebQTL*) an on-line resource to study complex gene networks and phenotypes [31].

**Figure 1:**
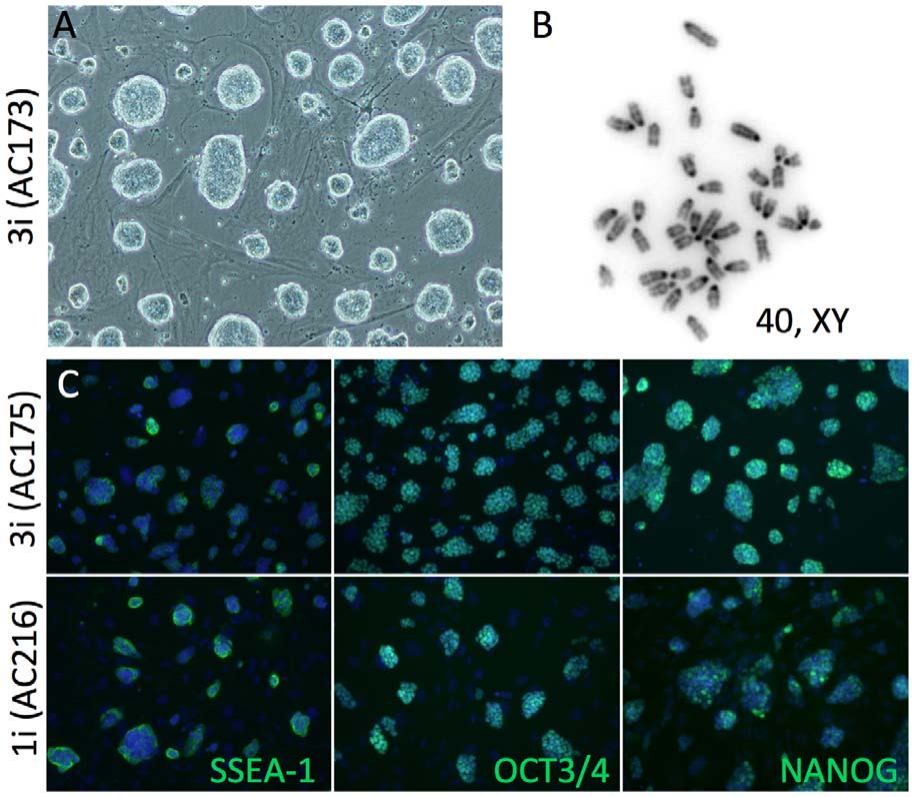
DBA/2J ES cell derivation. (**A**) In each round of derivation, 11–60% of embryos gave rise to stable embryonic stem cell lines (see also Table 1). (**B**) Approximately ∼20% of lines were male with normal chromosome counts, regardless of culture conditions. (C) All of the ES cell lines, regardless of culture conditions, expressed the essential pluripotency markers NANOG, SSEA-1 and OCT-3/4 (POU5F1).

**Figure 2:**
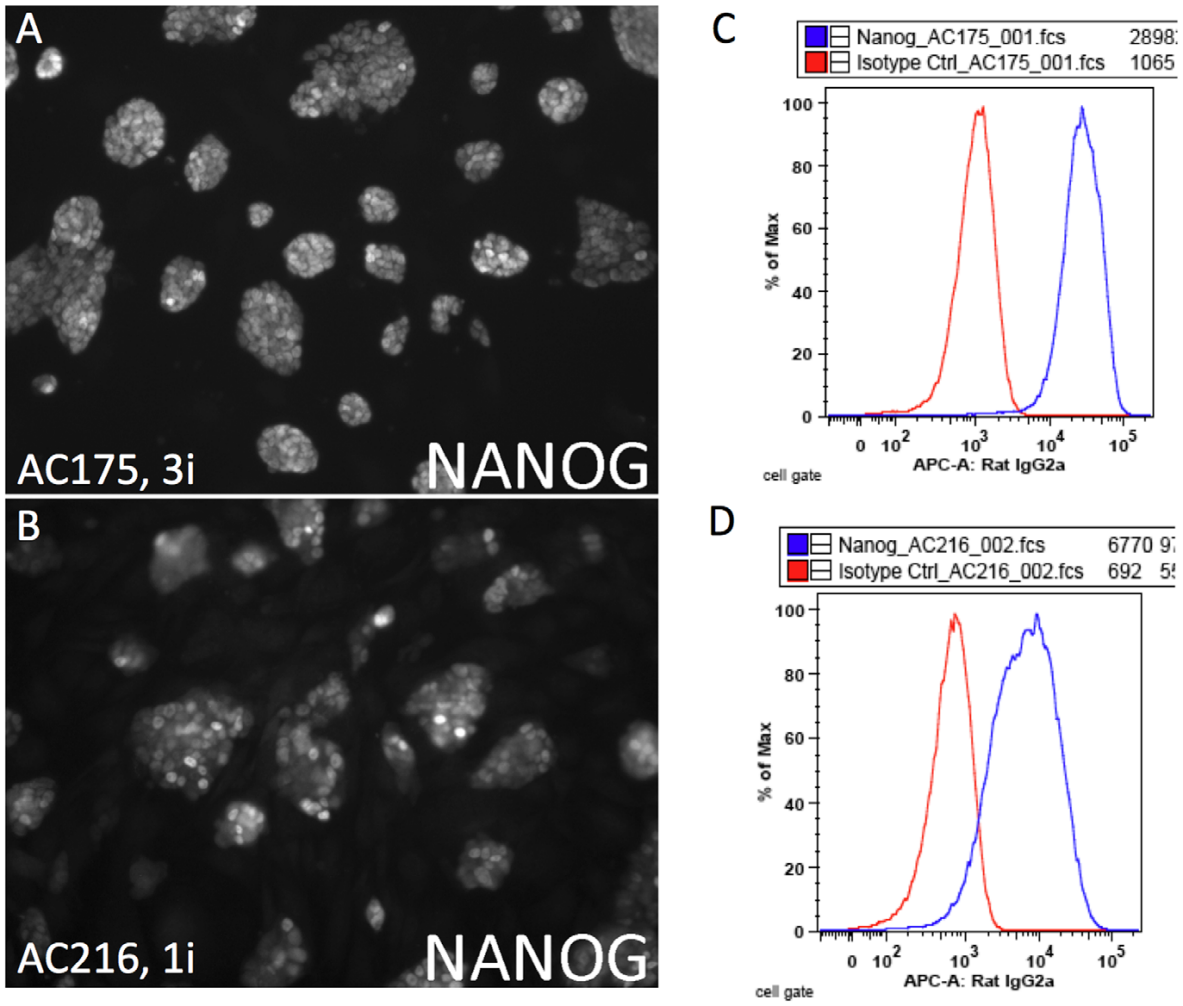
Variable NANOG expression in DBA/2J ES cell lines. (**A–D**) Representative images and FLOW analysis of 3i (AC175, A,C) and 1i lines (AC216, B,D) immunolabeled with an antibody to NANOG. Robust NANOG expression was characteristic of lines derived and cultured in 3i conditions.

**Table 1:** DBA/2J ES cell derivation.

Media conditions	Number of blastocysts	% blastocytes that gave ES cell lines (n)	Number of euploid, male ES cell lines
3i, derivation 1	36	22% (8)	0
3i, derivation 2	20	60% (12)	4
1i, derivation 1	36	11% (4)	1
1i derivation 2	20	60% (12)	4
1i, C57BL/6J	20	70% (14)	4

Two derivations of two different media types were used to derive DBA/2J ES cell (rows 1–4) and was compared to a control derivation using C57BL/6J blastocysts (row 5). Details are provided for: the total number of blastocyst attempted (column 2); the % of blastocysts that gave ES cell lines (column 3); and the total number of euploid male ES cell lines generated for each derivation (column 4).

Stable ES cell lines for gene targeting are available only for inbred strains from which stable ES cells lines can be consistently derived from the inner cell mass of blastocyst stage embryos. These permissive inbred strains include B6 and 129 derived strains [32], [33]. However, DBA/2J is one of many inbred strains that are considered recalcitrant to ES cell derivation because pluripotent embryonic stem cells (ES cells) are not easily derived [32], [33] from the inner cell mass of blastocyst stage embryos. Therefore, ES cell lines for gene targeting are simply not available. This severely hampers the ability to use DBA/2J mice to assess the role of specific genes in many human diseases. Previously, introducing mutant alleles into DBA/2J mice required targeting of the allele in an available ES cell line (usually B6- or 129-derived), followed by extensive backcrossing of the mutation to DBA/2J (for greater than 10 generations to replace the ES cell genome with a DBA/2J genome). Even after extensive backcrossing, a substantial interval of ES cell-derived DNA will remain surrounding the targeted gene, potentially confounding analysis of the phenotype.

**Figure 3:**
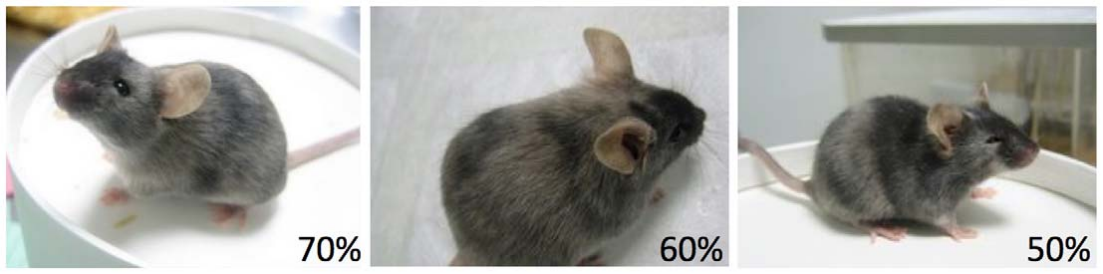
DBA/2J ES cell-derived male chimeras. DBA/2J ES cells were injected into C57BL/6J-derived blastocyst. Estimated percentage of coat derived from DBA/2J ES cells (dilute brown agouti) was used to determine the percent chimerism in each pup.

**Table 2:** Chimerism and germ line transmission of DBA/2J ES cells.

Cell line, culture condition	% of chimeric mice born (n)	% male chimeras	% germline chimeras	transmission rate (male only)
AC203, 1i	16% (19)	67%	100%	100%
AC216, 1i	38% (13)	100%	20%	100%
AC173, 3i	59% (37)	68%	92%	97%
AC175, 3i	46% (13)	100%	67%	100%

Four DBA/2J ES cell lines (two derived from each media type, 1i and 3i) were assessed for capacity to contribute to the germ line. These data are based on standard microinjection of ES cells into C57BL/6J host blastocysts. Additional data, including laser assisted microinjection data can be found in Table S1. Details are provided for: the number of chimeric mice (determined based on coat showing at least partial dilute brown agouti color, column 2); the % of those chimeric mice that were male (column 3); the % of male chimeric mice that sired offspring with 100% dilute brown agouti color (column 4); and the % of offspring that were completely dilute brown agouti (transmission rate, column 5).

Previous attempts to derive ES cell lines from DBA/2J mice have failed and therefore the DBA/2J strain is considered to be recalcitrant to ES cell derivation [33]. However, recent advances in stem cell biology have revealed that embryonic stem cells derived from the inner cell mass of some strains and some species (like the laboratory rat) have a limited capacity to maintain pluripotency and self-renewal in culture without consistent inhibition of signaling pathways that promote early differentiation [34], [35]. Therefore, addition of small molecule inhibitors of fibroblast growth factor (FGF)/mitogen-activated protein kinase (MEK)/extracellular signal-related kinase pathways and of glycogen synthase kinase 3 (Gsk3) have proven highly effective for successful derivation of ES cells from rats and from a recalcitrant mouse strain (NOD) [36]. Taking advantage of this renewed understanding of ES cell pluripotency, we surmised that recalcitrance in DBA/2J is likely due to the same underlying differentiation pathways. By inhibiting these pathways we successfully derived germ line competent ES cells. Using these cells, we have generated the first engineered allele in the DBA/2J genome.

## Methods

### Mouse Strains, Breeding and Husbandry

All DBA/2J mice used in this study were obtained from either The Jackson Laboratory production facility (Bar Harbor, ME) or from our research colony. Our DBA/2J colony is routinely crossed with DBA/2J mice from The Jackson Laboratory production facility to prevent genetic drift. Mice were housed in a 14 h light to 10 h dark cycle under previously described conditions [37]. The Jackson Laboratory Animal Care and Use Committee approved all of the experiments in this study.

**Figure 4:**
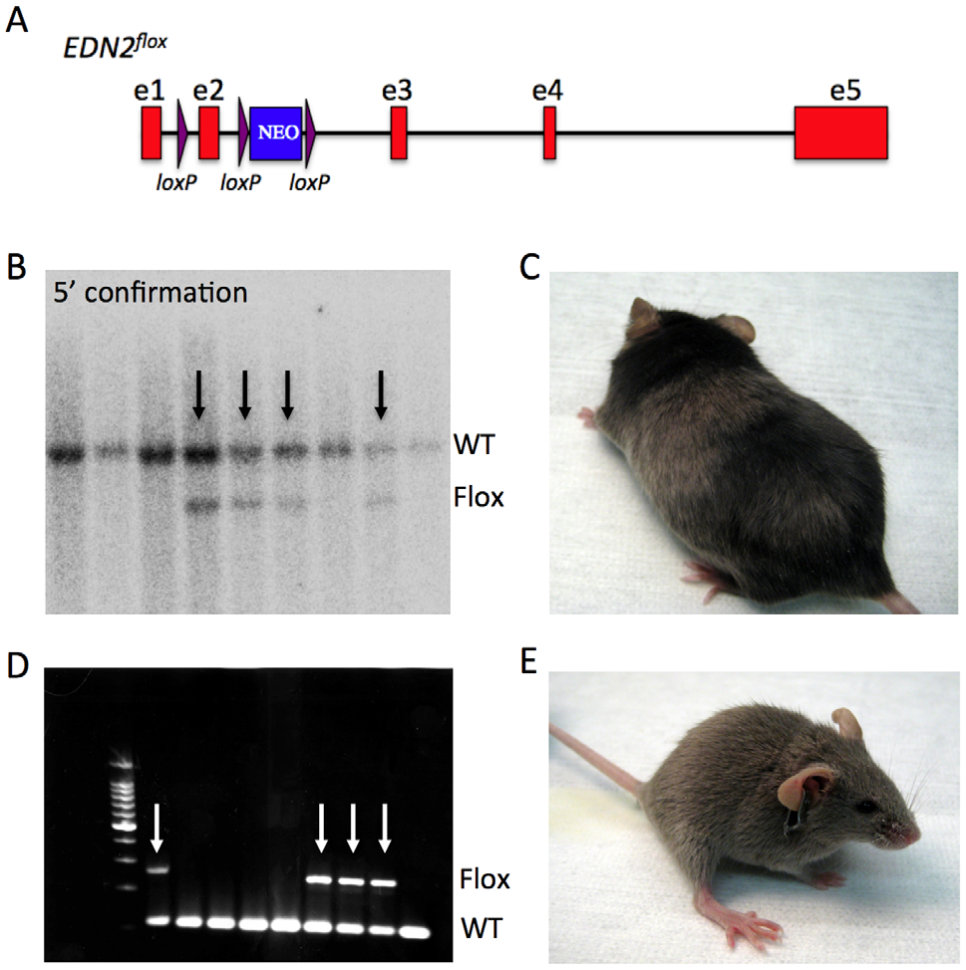
Generating a conditional allele of *Edn2* directly in DBA/2J-derived ES cells. (**A**) A targeting construct was generated that included *loxP* sites flanking exon 2 (e2) of the *Edn2* gene. Exon 2 contains part of the key functional domain for Edn2, and removal of exon 2 induces a frameshift for protein translation (**B**) Correct targeting at the 5′ end of the Edn2 gene was assessed using Xba1 restriction digests and southern blotting. The presence of the Neomycin (NEO) selectable marker incorporates an additional *Xba*1 restriction enzyme site in the targeted allele, generating a 6.6 kb fragment (Flox) compared to the normal 8.7 kb fragment (WT). Black arrows indicate clones correctly targeted at the 5′ end. (**C**) An example of a chimeric mouse generated as a result of injection of targeted DBA/2J ES cell clones into C57BL/6J blastocysts. (**D–E**) Chimeric male mice were mated with DBA/2J females and offspring genotyped for the heterozygous presence of the loxP sites in intron 1 (WT = 481 bp, Flox = 631 bp, see Methods). White arrows indicate mice that carry the floxed allele of *Edn2*. (D). See methods for primer information. As expected, 100% of heterozygous mutant offspring had the DBA/2J coat color (dilute brown agouti, E).

### Generation of DBA/2J-derived ES Cells

#### Embryo harvest and inner cell mass (ICM) outgrowth

Embryos from natural DBA/2J matings were collected at 3.5 days post coitum by flushing uteri with M2 medium (Millipore). Blastocysts were transferred directly to MEF feeder layers (cell cycle arrested mouse embryonic fibroblasts) in 4-well IVF (in vitro fertilization) culture dishes, 1–2 blastocysts per well. Morulae were cultured overnight in KSOM/M16 (Millipore) medium and the resulting blastocysts were transferred to MEF feeder layers the following day. Blastocysts were then cultured at 37°C, 5% CO_2_ for 8–10 days. During the first 4 days, blastocysts were undisturbed to allow for zona hatching and attachment of the embryos to the feeder layers. During days 5–10, the media were replaced every other day and inner cell mass (ICM) outgrowth was observed. The media conditions of ICM outgrowth were either ‘1i outgrowth media’ or ‘3i media’.

#### ES cell media

DMEM *(*Dulbecco’s Modified Eagle’s Medium, 4500 mg/L glucose and sodium bicarbonate, without L-glutamine and sodium pyruvate, Sigma), 15% fetal calf serum (Lonza), penicillin (100 units)/streptomycin (100 µg) (Life Technologies), Glutamax (1X, Life Technologies), 0.1 mM beta-mercaptoethanol, non-essential amino acids (1X, Life Technologies), sodium pyruvate (1 mM, Life Tecnologies), leukemia inhibitory factor (LIF, ESGRO 1000 U/ml, Chemicon).

#### 1i outgrowth media

ES cell media (as described above) containing KOSR (knockout serum replacement, Life Technologies) in place of fetal calf serum, and PD98059 (MAP kinase inhibitor, 50 µM, Calbiochem [32]) with LIF (1000 U/ml).

#### 3i media

serum free media (as described in [34]] containing 3 inhibitors (PD17304 (FGF receptor tyrosine kinase inhibitor, 100 nM, Stemgent), PD0325901 (MEK kinase inhibitor, 1 µM, Stemgent) and CHIR99021 (glycogen synthase kinase-3 inhibitor, 3 µM)), and LIF (leukemia inhibitory factor, ESGRO 1000 U/ml, Chemicon).

#### Disaggregation and culture

After 8–10 days, each outgrowth was given a unique identification number (AC###) and mechanically disaggregated. Disaggregated fragments were plated onto new MEF feeder layers in the same media type used for outgrowth (except for 1i media where KOSR was replaced with serum). After ∼2 days of growth, each emergent cell line was enzymatically dissociated using 0.05% trypsin EDTA (Life Technologies) and re-plated (P1). Light microscopy was used to monitor cell dissociation and trypsin exposure time was adjusted accordingly (usually 2–4 minutes depending on the culture conditions and cell line).

Emergent cell lines grown in 3i serum free media required less exposure (∼2 minutes) to 0.05% trypsin for dissociation and trypsin inhibition (using an equal volume of defined trypsin inhibitor, DTI, Life Technologies). Each cell line was expanded to P4 and any lines with overt differentiation or poor growth were discarded.

### ES Cell Line Characterization

#### Karyotypic analysis

ES cells were grown in 60 mm culture dishes until sub-confluent. Approximately 1–2 hours after media replacement, colcemid (0.02 mg/ml) was added to the media and the cells were incubated at 37°C, 5% CO_2_ for three hours. Cells were trypsinized, re-suspended by slow addition 0.56% KCl with constant agitation and incubated for 30–60 minutes. Cells were then fixed with ice-cold 3∶1 methanol:glacial acetic acid. Fixed cell suspensions were added dropwise to the surface of clean, moist glass slides and slides were air dried at 37°C for 1–2 days. Slides were mounted with Vectashield® hard set mounting reagent with DAPI (4′, 6-diamidino-2-phenylindole) (Vector Laboratories). A Spectral Karyotyping Microscope (SKY, Applied Spectral Imaging, Ltd.) was used to automatically scan slides for mitotic chromosome spreads and images were digitally captured. Chromosomes were counted in at least 40 well-spread, well-isolated nuclei and sex chromosomes were identified by morphology and distribution of heterochromatin (as assessed by DAPI, which preferentially binds A-T rich DNA).

#### Immunolabeling

Sub-confluent ES cells were fixed with 4% paraformaldehyde (PFA) for 20 minutes and washed 3×5 minutes with phosphate buffered saline (PBS). Fixed cells were then blocked with 5% fetal bovine serum (FBS), 0.1% triton-X for 15 minutes, incubated with primary antibody for 4 hours and washed 3×5 minutes with PBS. Secondary antibody incubations were 2 hours, and subsequent washes were 3×5 minutes with PBS, incubation with DAPI (2 mg/ml) for 10 minutes and 2×5 minute washes with PBS. Primary antibodies were anti-OCT 3/4 (1∶250, Santa Cruz Biologicals, #sc-5279), anti-NANOG (1∶250, Abcam, #ab21603) and anti-SSEA-1 (1∶250, Santa Cruz Biologicals, #sc-21702). Secondary antibodies were goat anti-mouse IgG Alexa Fluor® 488 (1∶1000, Life Technologies) and goat anti-rabbit IgG Alexa Fluor® 488 (1∶1000, Life Technologies). All antibodies were diluted with PBS, 1% FBS. All incubations and washes were performed at room temperature with gentle rocking. Labeled cells were visualized and images were collected with a Zeiss Axio Observer Z1 inverted microscope and a Zeiss AxioCam CCD camera.

To prepare and immunolabel ES cells for flow cytometry, cells were washed, counted and fixed in 4% PFA (100 µl per 1×10^∧^6 cells) for 15 minutes at room temperature. Cells were then washed 2×5 minutes in PBS, 1% FBS and stored in 1 ml aliquots at 1×10^∧^6 cells/ml for up to 72 hours at 4°C. For antibody labeling, cells were transferred to 12×75 mm polypropylene tubes, permeabilized for 15 minutes (Perm/Wash Buffer, BD Biosciences), incubated with mouse Fc block (purified rat IgG_2b_ anti-mouse CD16/CD32 monoclonal antibody, BD Biosciences) for 15 minutes, split and then incubated with either rat anti-Nanog Alexa Fluor® 647 (50 µg/ml, eBioscience) or rat IgG2a isotype control Alexa Fluor® 647 (eBioscience). Post-labeling washes were 2×5 minutes in PBS, 1% FBS and cells were resuspended in 400 µl of PBS, 1% FBS. Immunolabeled cells were then analyzed using a FACSCalibur flow cytometer (BD Biosciences).

### Microinjection and Chimera Production for Parental ES Cell Lines

Each parental ES cell line (AC203, AC216, AC173 and AC175) was tested for germ line competence by microinjection. For each line, ∼12 ES cells were microinjected into 50 C57BL/6J (black, *a/a*, The Jackson Laboratory stock #00664) host blastocysts and 6 ES cells were injected into 8 cell C57BL/6J embryos by the laser assisted method using a Hamilton Thorne XYClone. Male coat color chimeras were generated from both injection methods. Male chimeras were bred to DBA/2J females (dilute brown, *a*/*a Tyrp1^b^*/*Tyrp1^b^ Myo5a^d^*/*Myo5a^d^,* The Jackson Laboratory stock #000671) and germ line transmission of the DBA/2J ES cell line was assessed by calculating the % dilute brown offspring over three litters for each fertile male chimera.

### Generation of EDN2 Targeting Construct, pMCS-EDN2-DTA

A Frt-Loxp (FL) was inserted in intron 1 and a LNL (Loxp-Neo-Loxp) cassette was inserted in intron 2 of *Edn2* gene on a BAC clone (RP23-265J18) by BAC recombineering so that exon 2 of *Edn2* gene was flanked by two loxp sites. A gene targeting vector, pMCS-EDN2-DTA, was constructed by retrieving a 10 kb long homology arm (5′ to FL), LNL, and a 2 kb short homology arm (end of LNL to 3′) into a plasmid vector carrying the DTA (Diphtheria toxin alpha chain) negative selection marker.

### Targeting DBA/2J ES Cells with *Edn2* Conditional Knockout Construct

Prior to targeting, the following parameters for DBA/2J ES cell line AC173 were established; geneticin sensitivity (150 µg/mL), required working concentration of trypsin inhibitor (0.25 mg/mL), and compatibility of serum free media with available MEF stocks (irradiated at passage 3 or lower). In preparation for electroporation, pMCS-EDN2-DTA was prepared using a mini-prep kit (Qiagen) and DNA linearized with *Not*I. Electroporation was performed using the BTX ECM 830 electroporator. Ten million DBA/2J ES cells (line AC173, P9) were electroporated with 20 µg of DNA at a concentration of 3.8 µg/uL. Cells were plated onto 60 mm dishes in 3i media. Selection with geneticin at 150 µg/mL was started the following day and continued for 9 days. Plates were then switched back to media not containing selection antibiotic until day 12 post electroporation. On day 12, colonies from half the 60 mm plates were picked, selecting each independent colony into a separate 96 well. Colonies from the remaining 60 mm plates were picked on day 13 for a total of 46 colonies. Clones were expanded into two sets of 96 well plates to allow for a stock freeze of one set of 96 well plates and lysate preparation for their duplicates for Southern blot screening. Clones were maintained on MEFs due to poor growth of the DBA/2J line in the absence of MEFs. A total of 43 clones were frozen.

### Confirmation of Correct Targeting by Southern Blotting

For each of the 43 clones, Southern blotting was used to assess correct targeting of the 5′ end of the *Edn2* locus. A 622bp 5′ probe was generated upstream of the *Edn2* gene (forward primer, GATGTGGGTGGCCAGTGGC; reverse primer, GCTGGGAGCAGAGGCCAGGTG). DNA from each of the 43 clones was digested with *Xba*1. Correct integration of the targeting construct introduced a novel *Xba*I site to generate a 6.6 kb band after digestion compared to an 8.7 kb band for non-targeted clones. Five of the 43 clones were correctly targeted on the 5′ end (clones 5, 32, 33, 34, and 36), a targeting efficiency of approximately 11.5% of tested clones. For these five clones, Southern blotting was again used to assess correct targeting at the 3′ end. A 1001 bp 3′ probe was generated by PCR (forward primer, AGTAGACAGTGGACAGTTCC; reverse primer, CAAAAGTGTCCCAAGAGAACAGT). Correct integration of the targeting construct at the 3′ end disrupted an *Eco*RI site to generate a 16.2 kb band after digestion compared to a 15.2 kb band for non-targeted clones. Clones 33 and 36 were identified as properly targeted on both ends. (All primers shown 5′ to 3′).

### Microinjection of Targeted Clones and Confirmation of Germ Line Transmission

For microinjection of EDN2-targeted AC173 ES cell lines, the correctly targeted clones (33 and 36) were thawed from 96 well plates to 12 well MEF plates using 3i media and injected into C57BL/6J blastocysts (∼12 ES cells per embryo). Both clones were at passage 22 at the time of injection. Passage number indicates the total number of trypsin exposures from the emergence of the ES cell line (see *Disaggregation and culture* above). In total, 149 injected blastocysts (approximately half from each of clone 33 and 36) were transferred into 13 pseudopregnant B6(Cg)-*Tyr*<c-2J>/J recipients (albino). For clone 33, 3 chimeric pups were produced (all male) and for clone 36, 2 chimeric pups (1 male and 1 female). Percentage chimerism ranged from 20–50% for both clones (based on coat color).

### Mouse Genotyping

To determine the presence or absence of the *EDN2^flox^* allele, *Edn2*-Intron2-F (CATAGAGCGGTGAGGCCACAG) and *Edn2*-Intron2-R: (AAGTTGGCACCCTTGGTGTTC) primers were used. The primers amplify a region of intron 2 that includes the first *loxP* site (*Edn2^+^* allele, 113bp; *Edn2^flox^* allele, 259bp).

## Results

### DBA/2J ES Cell Derivation

To define the most efficient protocol for derivation of ES cell lines from DBA/2J, we tested two media conditions that have been previously shown to promote ES cell derivation efficiency and ES cell pluripotency for other recalcitrant mouse strains. These media were tested in parallel over two rounds of derivation. The first medium, ‘1i’, was standard mouse ES cell medium, including LIF (leukemia inhibitory factor) and a single inhibitor of MAP kinase/ERK kinase, PD98059. This medium was previously shown to promote ES cell derivation efficiency from the recalcitrant CBA mouse strain [32]. More recently, several laboratories have had great success deriving ES cells not only from recalcitrant mouse strains (e.g. NOD), but also recalcitrant species (rat) by using defined serum free culture conditions combined with inhibitors of MEK kinase, FGF receptor tyrosine kinase and glycogen synthase kinase-3 [34], [35], [38]. Therefore, the second media condition, ‘3i’ was serum free media, including LIF and 3 inhibitors, PD17304 (FGF receptor tyrosine kinase inhibitor), PD0325901 (MEK kinase inhibitor, 1 µM, Stemgent) and CHIR99021 (glycogen synthase kinase-3 inhibitor) [34]. Finally, to promote growth of the inner cell mass and to minimize trophoblast differentiation, FBS was excluded from the 1i media during the inner cell mass (ICM) outgrowth stage.

### Comparing Derivation Media

ES cell lines were derived from a total of 112 DBA/2J embryos over 2 rounds of derivation (derivation 1 and derivation 2) as shown in Table 1. Regardless of the media used, ES cell derivation efficiency (number of established ES cell lines/number of embryos) was comparable to permissive strains like C57BL/6J and significantly better than has been previously reported for DBA/2J, 1–6% [33]. From a total of 36 ES cell lines, 9 were euploid (>70% normal chromosome counts over 40 nuclei) male lines. These 9 lines were further characterized to select a subset of lines for germ line testing (Figure 1). All 9 ES cell lines showed expression of alkaline phosphatase and the pluripotency markers NANOG, SSEA-1 and OCT 3/4 (POU5F1), however ES cell lines that were derived and cultured in 1i medium showed variable expression of NANOG compared to ES cell lines derived and cultured in 3i medium. This variable expression was evident both qualitatively by immunofluorescence microscopy and quantitatively by flow cytometry, where a broad range of NANOG expression was consistently present in 1i cultures, including a fraction of ES cells with NANOG expression as low as background (Figure 2).

### Assessing Chimerism and Germ Line Efficiency

The capacity of an ES cell line to contribute to the germ line of a chimeric animal is the gold standard test of pluripotency for mouse ES cell lines and a pre-requisite for any ES cell line to be used to generate genetically engineered mice. Four DBA/2J ES cell lines were selected for germ line testing based on media conditions (two 1i ES cell lines and two 3i ES cell lines), NANOG expression, and chromosome counts (those with the highest percent of euploid nuclei were favored). These were AC203 (1i, 86% euploid), AC216 (1i, 86% euploid), AC175 (3i, 78% euploid) and AC173 (3i, 86% euploid). All four ES cell lines successfully contributed to chimeras and to the germ line, as determined by coat color (Table 2, Figure 3). The 2 ES cell lines derived and grown in 3i conditions (AC175 and AC173) produced larger percentages of coat color chimeras upon injection into C57BL/6J host embryos, consistent with the more robust expression of the pluripotency factor NANOG in these ES cell lines (Table S1).

### Testing Gene Targeting Efficiency in DBA/2J ES Cells

The DBA/2J strain is a widely used mouse model of glaucoma. Glaucoma is characterized by the degeneration of retinal ganglion cells and the degeneration of the optic nerve. We have shown previously that Endothelin-2 (*Edn2*), a component of the endothelin system is upregulated in monocytes and microglia-like cells in the retina and optic nerve during glaucoma [4], [5]. Given that no conditional allele was available for *Edn2* on a DBA/2J background, *Edn2* was selected as the gene of interest to assess targeting capabilities for the newly derived DBA/2J ES cells. A targeting construct was generated and used to target the AC173 ES cell line (one of the parental lines with robust NANOG expression and a high degree of chimerism in earlier tests, Figure 4). After targeting and subsequent colony expansion, 43 clones were assessed for correct targeting of the *Edn2* locus. Five of the 43 clones (11%) were correctly targeted on the 5′ end, two of which were also correctly targeted on the 3′ end. These two clones (*Edn2*-clone33 and *Edn2*-clone36) were injected into C57BL/6J blastocysts and chimeric pups obtained. Chimerism for mice derived from either clone ranged from 20–50% (Table S1). Male chimeric mice were bred to DBA/2J females and approximately 50% of offspring were heterozygous for the conditional *Edn2* allele. More than 20 mice heterozygous have been aged for 6–12 months. They have a healthy appearance with no gross adverse phenotypes or premature death.

## Discussion

We have demonstrated that ES cells can be derived from DBA/2J mice and that these ES cells are competent to contribute to the germ line both at low passages and higher passages, post-targeting. Moreover, we report the first use of these DBA/2J ES cells to generate a genetically engineered mouse strain. While both media conditions used to derive DBA/2J ES cells favored successful derivation of germ line competent cells, serum free 3i media promoted more robust NANOG expression and higher percentages of coat color chimeras. This is likely due to inhibition of FGF signaling through more efficient inhibition of phospho-ERK combined with the restorative effects of glycogen synthase kinase inhibition [34]. A recent study indicates that inhibition of the FGF receptor tyrosine kinase through PD173074 is not necessary in combination with PD0325901, hence 2i media was equally effective for maintenance of pluripotency in ES cells [39]. Efficient inhibition of FGF signaling promotes the pluripotent ground state of embryonic stems cells, the hallmark of which is NANOG expression [40].

Serum free, 3i culture conditions are frequently used in the absence of MEFs feeder layers since the leukemia inhibitory factor provided by the MEF’s can instead be added to the culture medium. However, we found that in the absence of feeders, emergent ES cell lines in serum free, 3i medium were loosely adherent, despite the use of gelatin-coated plates. This made them subsequently difficult to manipulate during gene targeting. Therefore, MEF feeder layers were used throughout our derivation, culture and targeting of DBA/2J ES cells regardless of the culture media used. We found that use of low passage MEFs that have been previously screened for tolerance to serum free culture was critical for maintenance of our serum free, 3i DBA/2J ES cells, especially during drug selection post-targeting where feeder layers are exposed, long term to serum free culture conditions.

The availability of DBA/2J ES cells will enable genetic manipulation of genes of interest directly on a DBA/2J background. As we have shown with the *Edn2* gene, it is possible to generate conditional alleles without the need for costly and time-consuming backcrossing from other genetic backgrounds (commonly C57BL/6J or 6N). Targeting constructs for many genes are available through the Knockout Mouse Project. Although many constructs were generated using C57BL/6N, many will be useful for generation of conditional alleles directly in DBA/2J ES cells. This will greatly aid in the ability to test the role(s) of specific genes of interest. Importantly, this ability means that no congenic interval that is derived from the original strain and that can confound experiments will surround the targeted gene.

In summary, the derivation of ES cells from DBA/2J mice will accelerate the ability to generate genetically engineered DBA/2J mice. These resources will aid in the understanding of and the development of improved therapies for a wide variety of human diseases including glaucoma, deafness and schizophrenia.

## Supporting Information

Table S1 **Additional chimerism and germ line data transmission data, including laser assisted microinjection data.**
(XLSX)

## References

[1] Anderson MG, Smith RS, Hawes NL, Zabaleta A, Chang B, et al (2002) Mutations in genes encoding melanosomal proteins cause pigmentary glaucoma in DBA/2J mice. Nat Genet 30: 81–85. 11743578 10.1038/ng794

[2] Bosco A, Inman DM, Steele MR, Wu G, Soto I, et al (2008) Reduced retina microglial activation and improved optic nerve integrity with minocycline treatment in the DBA/2J mouse model of glaucoma. Invest Ophthalmol Vis Sci 49: 1437–1446. 18385061 10.1167/iovs.07-1337

[3] Calkins DJ, Horner PJ, Roberts R, Gradianu M, Berkowitz BA (2008) Manganese-enhanced MRI of the DBA/2J mouse model of hereditary glaucoma. Invest Ophthalmol Vis Sci 49: 5083–5088. 18552381 10.1167/iovs.08-2205 PMC2586056

[4] Howell GR, Macalinao DG, Sousa GS, Walden M, Soto I, et al (2011) Molecular clustering identifies complement and endothelin induction as early events in a mouse model of glaucoma. Journal of Clinical Investigation 121: 1429–1444. 21383504 10.1172/JCI44646 PMC3069778

[5] Howell GR, Soto I, Zhu X, Ryan M, Macalinao DG, et al (2012) Radiation treatment inhibits monocyte entry into the optic nerve head and prevents neuronal damage in a mouse model of glaucoma. The Journal of clinical investigation 122: 1246–1261. 22426214 10.1172/JCI61135 PMC3314470

[6] Inman DM, Horner PJ (2007) Reactive nonproliferative gliosis predominates in a chronic mouse model of glaucoma. Glia 55: 942–953. 17457855 10.1002/glia.20516

[7] Libby RT, Anderson MG, Pang IH, Robinson ZH, Savinova OV, et al (2005) Inherited glaucoma in DBA/2J mice: pertinent disease features for studying the neurodegeneration. Vis Neurosci 22: 637–648. 16332275 10.1017/S0952523805225130

[8] May CA, Mittag T (2006) Optic nerve degeneration in the DBA/2NNia mouse: is the lamina cribrosa important in the development of glaucomatous optic neuropathy? Acta Neuropathol (Berl) 111: 158–167. 16453144 10.1007/s00401-005-0011-2

[9] Nagaraju M, Saleh M, Porciatti V (2007) IOP-dependent retinal ganglion cell dysfunction in glaucomatous DBA/2J mice. Invest Ophthalmol Vis Sci 48: 4573–4579. 17898280 10.1167/iovs.07-0582 PMC2031015

[10] Panagis L, Zhao X, Ge Y, Ren L, Mittag TW, et al (2009) Gene Expression Changes in Areas of Focal Loss of Retinal Ganglion Cells (RGC) in the Retina of DBA/2J Mice. Invest Ophthalmol Vis Sci 51: 2024–2034. 19737878 10.1167/iovs.09-3560 PMC2868411

[11] Schlamp CL, Li Y, Dietz JA, Janssen KT, Nickells RW (2006) Progressive ganglion cell loss and optic nerve degeneration in DBA/2J mice is variable and asymmetric. BMC Neurosci 7: 66. 17018142 10.1186/1471-2202-7-66 PMC1621073

[12] Schuettauf F, Rejdak R, Walski M, Frontczak-Baniewicz M, Voelker M, et al (2004) Retinal neurodegeneration in the DBA/2J mouse-a model for ocular hypertension. Acta Neuropathol (Berl) 107: 352–358. 14745571 10.1007/s00401-003-0816-9

[13] Chung SH, Johnson MS (1984) Studies on sound-induced epilepsy in mice. Proceedings of the Royal Society of London Series B, Containing papers of a Biological character Royal Society 221: 145–168. 10.1098/rspb.1984.0028 6145159

[14] Connolly PM, Maxwell CR, Kanes SJ, Abel T, Liang Y, et al (2003) Inhibition of auditory evoked potentials and prepulse inhibition of startle in DBA/2J and DBA/2Hsd inbred mouse substrains. Brain research 992: 85–95. 14604776 10.1016/j.brainres.2003.08.035

[15] Alberts R, Srivastava B, Wu H, Viegas N, Geffers R, et al (2010) Gene expression changes in the host response between resistant and susceptible inbred mouse strains after influenza A infection. Microbes and infection/Institut Pasteur 12: 309–318. 10.1016/j.micinf.2010.01.008 20114087

[16] Drake TA, Schadt E, Hannani K, Kabo JM, Krass K, et al (2001) Genetic loci determining bone density in mice with diet-induced atherosclerosis. Physiol Genomics 5: 205–215. 11328966 10.1152/physiolgenomics.2001.5.4.205

[17] Nedelko T, Kollmus H, Klawonn F, Spijker S, Lu L, et al (2012) Distinct gene loci control the host response to influenza H1N1 virus infection in a time-dependent manner. BMC genomics 13: 411. 22905720 10.1186/1471-2164-13-411 PMC3479429

[18] Paigen B (1995) Genetics of responsiveness to high-fat and high-cholesterol diets in the mouse. Am J Clin Nutr 62: 458S–462S. 7625360 10.1093/ajcn/62.2.458S

[19] Willott JF, Bosch JV, Shimizu T, Ding DL (2006) Effects of exposing DBA/2J mice to a high-frequency augmented acoustic environment on the cochlea and anteroventral cochlear nucleus. Hear Res 216–217: 138–145. 10.1016/j.heares.2006.01.010 16497456

[20] Philip VM, Duvvuru S, Gomero B, Ansah TA, Blaha CD, et al. (2009) High-throughput behavioral phenotyping in the expanded panel of BXD recombinant inbred strains. Genes Brain Behav. 10.1111/j.1601-183X.2009.00540.x PMC2855868 19958391

[21] Pichard C, Gorwood PA, Hamon M, Cohen-Salmon C (2009) Differential effects of free versus imposed motor activity on alcohol consumption in C57BL/6J versus DBA/2J mice. Alcohol 43: 593–601. 20004337 10.1016/j.alcohol.2009.10.007

[22] Wang X, Chen Y, Lu L Genetic regulatory network analysis for app based on genetical genomics approach. Exp Aging Res 36: 79–93. 20054728 10.1080/03610730903418729

[23] Davis RC, Schadt EE, Smith DJ, Hsieh EW, Cervino AC, et al (2005) A genome-wide set of congenic mouse strains derived from DBA/2J on a C57BL/6J background. Genomics 86: 259–270. 16039824 10.1016/j.ygeno.2005.05.010

[24] Anderson MG, Smith RS, Savinova OV, Hawes NL, Chang B, et al (2001) Genetic modification of glaucoma associated phenotypes between AKXD-28/Ty and DBA/2J mice. BMC genetics 2: 1. 11178107 10.1186/1471-2156-2-1 PMC29081

[25] Gilbert DJ, Neumann PE, Taylor BA, Jenkins NA, Copeland NG (1993) Susceptibility of AKXD recombinant inbred mouse strains to lymphomas. Journal of virology 67: 2083–2090. 8383230 10.1128/jvi.67.4.2083-2090.1993 PMC240292

[26] Boughter JD Jr, Mulligan MK, St John SJ, Tokita K, Lu L, et al (2012) Genetic Control of a Central Pattern Generator: Rhythmic Oromotor Movement in Mice Is Controlled by a Major Locus near Atp1a2. PloS one 7: e38169. 22675444 10.1371/journal.pone.0038169 PMC3364982

[27] Hsu HC, Lu L, Yi N, Van Zant G, Williams RW, et al (2007) Quantitative trait locus (QTL) mapping in aging systems. Methods in molecular biology 371: 321–348. 17634591 10.1007/978-1-59745-361-5_23

[28] Jellen LC, Beard JL, Jones BC (2009) Systems genetics analysis of iron regulation in the brain. Biochimie 91: 1255–1259. 19393285 10.1016/j.biochi.2009.04.009 PMC2742566

[29] McKnite AM, Perez-Munoz ME, Lu L, Williams EG, Brewer S, et al (2012) Murine gut microbiota is defined by host genetics and modulates variation of metabolic traits. PloS one 7: e39191. 22723961 10.1371/journal.pone.0039191 PMC3377628

[30] Mountz JD, Van Zant GE, Zhang HG, Grizzle WE, Ahmed R, et al (2001) Genetic dissection of age-related changes of immune function in mice. Scandinavian journal of immunology 54: 10–20. 11439143 10.1046/j.1365-3083.2001.00943.x

[31] Chesler EJ, Lu L, Wang J, Williams RW, Manly KF (2004) WebQTL: rapid exploratory analysis of gene expression and genetic networks for brain and behavior. Nature neuroscience 7: 485–486. 15114364 10.1038/nn0504-485

[32] Buehr M, Smith A (2003) Genesis of embryonic stem cells. Philos Trans R Soc Lond B Biol Sci 358: 1397–1402; discussion 1402. 10.1098/rstb.2003.1327 PMC1693233 14511487

[33] Gardner RL, Brook FA (1997) Reflections on the biology of embryonic stem (ES) cells. Int J Dev Biol 41: 235–243. 9184330

[34] Ying QL, Wray J, Nichols J, Batlle-Morera L, Doble B, et al (2008) The ground state of embryonic stem cell self-renewal. Nature 453: 519–523. 18497825 10.1038/nature06968 PMC5328678

[35] Hanna J, Markoulaki S, Mitalipova M, Cheng AW, Cassady JP, et al (2009) Metastable pluripotent states in NOD-mouse-derived ESCs. Cell Stem Cell 4: 513–524. 19427283 10.1016/j.stem.2009.04.015 PMC2714944

[36] Nichols J, Jones K, Phillips JM, Newland SA, Roode M, et al (2009) Validated germline-competent embryonic stem cell lines from nonobese diabetic mice. Nat Med 15: 814–818. 19491843 10.1038/nm.1996

[37] Smith RS, Zabaleta A, Kume T, Savinova OV, Kidson SH, et al (2000) Haploinsufficiency of the transcription factors FOXC1 and FOXC2 results in aberrant ocular development. Hum Mol Genet 9: 1021–1032. 10767326 10.1093/hmg/9.7.1021

[38] Buehr M, Meek S, Blair K, Yang J, Ure J, et al (2008) Capture of authentic embryonic stem cells from rat blastocysts. Cell 135: 1287–1298. 19109897 10.1016/j.cell.2008.12.007

[39] Silva J, Barrandon O, Nichols J, Kawaguchi J, Theunissen TW, et al (2008) Promotion of reprogramming to ground state pluripotency by signal inhibition. PLoS Biol 6: e253. 18942890 10.1371/journal.pbio.0060253 PMC2570424

[40] Silva J, Nichols J, Theunissen TW, Guo G, van Oosten AL, et al (2009) Nanog is the gateway to the pluripotent ground state. Cell 138: 722–737. 19703398 10.1016/j.cell.2009.07.039 PMC3437554

